# COVID-19 in Pregnancy: Maternal, Neonatal, and Racial Disparities in a Local Cohort

**DOI:** 10.7759/cureus.106549

**Published:** 2026-04-06

**Authors:** Celeena R Remmers, Diana E Sanchez, Funminiyi A Ajayi

**Affiliations:** 1 Department of Obstetrics and Gynecology, University of Illinois College of Medicine Rockford, Rockford, USA; 2 Department of Obstetrics and Gynecology, Mercyhealth Javon Bea Hospital, Rockford, USA

**Keywords:** covid-19, maternal outcomes, neonatal outcomes, pregnancy, racial disparities

## Abstract

Background

COVID-19 during pregnancy has been linked to adverse maternal and neonatal outcomes. However, data describing these outcomes by trimester and within specific local populations remain limited. The influence of COVID-19 on racial disparities in pregnancy outcomes is also not well understood. Accordingly, we investigated trimester-specific maternal and neonatal outcomes, as well as racial disparities, in pregnancies complicated by COVID-19 in Rockford, Illinois.

Objective

To describe maternal and neonatal outcomes among women diagnosed with COVID-19 during pregnancy at Mercyhealth Javon Bea Hospital in Rockford, Illinois, and to compare outcomes by trimester of infection, race, and with county-level data.

Methods

We conducted a retrospective chart review of obstetric patients diagnosed with COVID-19 during pregnancy who delivered between January 1, 2019, and October 28, 2022. Maternal and neonatal outcomes were assessed by trimester of infection and race and compared with 2021 Winnebago County outcomes. Chi-square tests, Fisher’s exact tests, and logistic regression were applied with 95% confidence intervals.

Results

Among 198 women diagnosed with COVID-19 (201 newborns), 15 (7.6%) were diagnosed in the first trimester, 73 (36.9%) in the second, and 110 (55.6%) in the third. Third-trimester infection was associated with pneumonia (13/110, 11.8%; p=0.024). First-trimester infection showed higher odds of preterm birth (7/17, 41.2%; OR=3.83, 95% CI 1.28-11.45) and low birth weight (4/17, 23.5%; OR=11.24, 95% CI 3.04-41.55). No maternal deaths were observed within the study period. Compared with 2021 Winnebago County data, our cohort had higher rates of gestational diabetes (16.2% vs. 10.8%; p=0.02) and ICU admission (1.5% vs. 0.3%; p=0.01). Other outcomes, including cesarean delivery, overall preterm birth, NICU admission, and low birth weight, were similar.

Conclusion

This single-center cohort suggests that trimester of infection may be associated with different maternal and neonatal outcomes, with pneumonia more common after third-trimester infection and preterm birth and low birth weight more frequent after first-trimester infection.

## Introduction

COVID-19 has significantly affected maternal and neonatal health outcomes worldwide. Studies have shown that pregnant patients with COVID-19 are at increased risk of ICU admission, invasive ventilation, and maternal mortality compared with non-pregnant patients [1]. Associated complications include gestational hypertension, preeclampsia, gestational diabetes, polyhydramnios, and cesarean delivery [2-4]. Despite these findings, most research has not examined trimester-specific impacts.

Neonatal risks include preterm birth, premature rupture of membranes, NICU admission, and stillbirth [3,4]. A meta-analysis confirmed higher rates of preterm birth and NICU admission [1]. Data also show worsened outcomes during the Delta wave, including higher stillbirth and maternal mortality rates [5].

Structural disparities also impact maternal outcomes. Black women face three times higher maternal mortality rates than White women [6]. Emerging studies suggest that Black women had lower COVID-19 vaccination uptake and higher infection risk due to systemic barriers [7,8].

This study was designed to address several critical gaps. Specifically, we examined: (1) maternal outcomes by trimester of COVID-19 infection; (2) neonatal outcomes by trimester of maternal infection; (3) racial differences in maternal outcomes; and (4) maternal and neonatal outcomes at our institution compared with county-level data from Winnebago County. By incorporating these four perspectives, we aimed to provide comprehensive, locally relevant data to guide prenatal counseling, care, and equity-focused interventions.

We hypothesized that earlier gestational infection may be associated with adverse neonatal outcomes, whereas later gestational infection may be associated with increased maternal respiratory morbidity. Key outcomes of interest included pneumonia, preterm birth, and low birth weight, while additional maternal and neonatal outcomes were considered exploratory. Analyses of racial disparities included both clinical outcomes and differences in structural and social factors, such as vaccination status, insurance coverage, and substance use.

This study was previously presented as an abstract at the 2024 National Medical Association's Annual Convention and Scientific Assembly on August 3, 2024.

## Materials and methods

Study design and setting

This study was a retrospective chart review conducted at Mercyhealth Javon Bea Hospital in Rockford, Illinois. The study population included all obstetric patients whose pregnancies began after January 1, 2019, and who were diagnosed with COVID-19 during pregnancy, with deliveries occurring through October 28, 2022. Although some pregnancies were initiated in 2019, all included patients delivered after July 4, 2020. COVID-19 infection was confirmed by inpatient or outpatient diagnostic testing or by documented self-reported positive results. The date of COVID-19 diagnosis was defined as the date of the first positive SARS-CoV-2 test documented in the medical record. For patients with self-reported positive results, the date recorded in the clinical documentation was used when corroborated by provider notes or subsequent care documentation. Symptom onset was not used for trimester classification. Both symptomatic and asymptomatic patients were eligible for inclusion. Institutional Review Board approval was obtained prior to data abstraction.

Data collection

Data were abstracted from the electronic medical record system. Maternal demographic variables included age, race/ethnicity, educational attainment, insurance, rurality, and documented comorbidities. Clinical factors included gravidity, parity, history of cesarean delivery, reported substance use, and COVID-19 vaccination status. Variables were defined based on documentation in the electronic medical record. Trimester of COVID-19 diagnosis was categorized as first trimester (last menstrual period to 13 weeks and 6 days), second trimester (14 weeks to 27 weeks and 6 days), and third trimester (28 weeks to 40 weeks and 6 days). Gravidity and parity were recorded at the time of delivery. Insurance status was categorized as public (Medicaid or Medicare) or private, and rurality was determined by ZIP code using US Census definitions. Pre-pregnancy weight was defined as the weight at the initial prenatal visit or the most recent prior encounter, and postpartum weight as the weight recorded at or shortly after delivery. Additional maternal and neonatal outcomes, including gestational diabetes, hypertension, pneumonia, ICU admission, preterm birth (<37 weeks), and low birth weight (<2500 g), were identified based on clinician documentation and standard clinical diagnoses recorded in the medical record.

Vital statistics data for Winnebago County were obtained directly from the Illinois Department of Public Health (IDPH), Division of Health Data & Policy, Office of Policy, Planning & Statistics. These data were provided to our study team by the state Vital Statistician as county-level summary tables for the calendar year 2021. Because these data are part of IDPH’s internal vital records database and are not publicly published in a citable report, no formal bibliographic reference is available. Accordingly, the dataset is described within the text and referenced in the relevant tables, Methods, Results, and Discussion sections to ensure transparent attribution of the source. County-level data represented all recorded births in Winnebago County for 2021. Variable definitions were based on standard vital statistics reporting; however, exact alignment with hospital-level definitions could not be independently verified.

Outcomes

Maternal outcomes assessed included mode of delivery, total gestational weight gain, pneumonia, gestational diabetes, gestational hypertension or preeclampsia, polyhydramnios, ICU admission, venous thromboembolism, and maternal mortality. Neonatal outcomes included gestational age at delivery, birth weight, NICU admission, Apgar scores, stillbirth, and cord blood laboratory results. Standardized COVID-19 severity classifications (NIH or CDC criteria for mild, moderate, severe, and critical illness) were not applied in this study. Analyses were based on the presence or absence of COVID-19 infection during pregnancy, and specific clinical outcomes were evaluated individually rather than as part of a composite severity classification. For multiple gestations, maternal outcomes were analyzed at the patient level, while neonatal outcomes were analyzed at the individual infant level. Neonatal analyses did not account for clustering by mother.

Statistical analysis

Maternal and neonatal outcomes were compared across trimesters of COVID-19 diagnosis. Additional analyses compared outcomes by race and against county-level data from Winnebago County’s 2021 maternal outcomes (unpublished internal data obtained from the Illinois Department of Public Health). Statistical tests included chi-square, Fisher’s exact test, and regression models adjusted for maternal age, BMI, insurance, education, and vaccination status. Analyses were conducted using available data. Variables with missing data were retained in analyses using a separate “unknown” category for categorical variables. Analyses were performed using SAS OnDemand for Academics (SAS Institute Inc., Cary, North Carolina).

## Results

**Figure 1:**
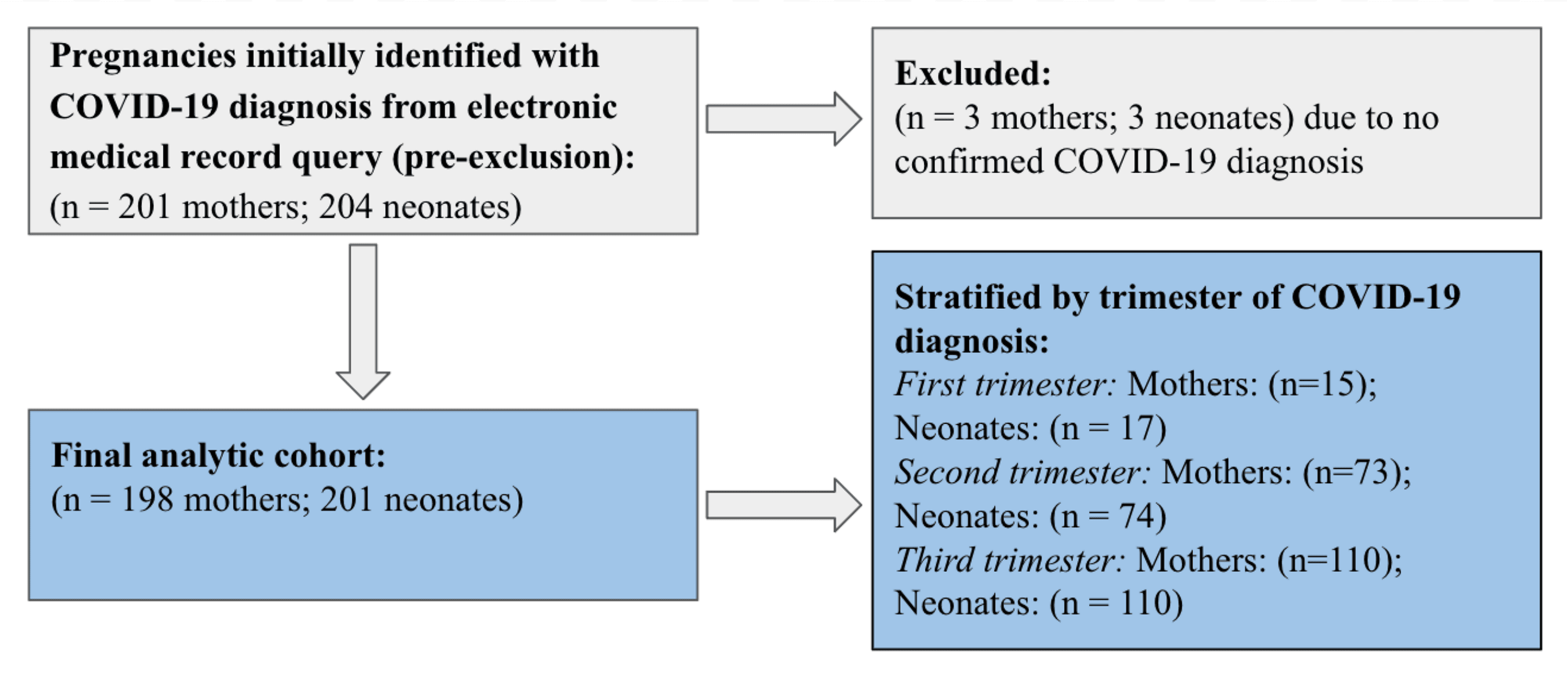
Study flow diagram of cohort selection Pregnancies with documented COVID-19 during pregnancy were identified through electronic medical record queries. After exclusion of cases without confirmed infection, the final analytic cohort included 198 mothers and 201 neonates. The difference between maternal and neonatal counts reflects multiple gestations. Neonates were stratified by trimester of maternal COVID-19 diagnosis.

A total of 198 pregnant women with COVID-19 delivered 201 newborns during the study period. Of these women, 15 (7.6%) were diagnosed in the first trimester, 73 (36.9%) in the second trimester, and 110 (55.6%) in the third trimester, after exclusion of cases without confirmed infection (Figure 1). The mean maternal age was 29 years, and most participants identified as White, with Black women representing approximately 11% of the cohort. Vaccination uptake decreased across trimesters, from 40% in the first trimester to 18% in the third trimester (Table 1).

**Table 1:** Characteristics of pregnant women by trimester of COVID-19 diagnosis This table summarizes demographic, socioeconomic, and health-related characteristics of pregnant women diagnosed with COVID-19 during the first, second, or third trimester. Categorical variables are presented as n (%) and continuous variables as mean ± SD. No statistically significant differences were observed across trimesters for education, race, ethnicity, insurance status, pre-pregnancy BMI, rurality, vaccination status, or substance use (tobacco, alcohol, or drug use). The majority of women were White (79–87%), non-Hispanic (73–80%), and resided in micropolitan areas (88–93%). Most participants had private insurance (57–73%), and pre-pregnancy BMI categories were distributed across normal, overweight, and obese ranges. Maternal age was similar across trimesters, with mean ages ranging from 28.7 to 30.7 years. P-values were calculated using chi-square or Fisher’s exact tests for categorical variables and analysis of variance for continuous variables.

Variables	First Trimester (n=15)		Second Trimester (n=73)		Third Trimester (n=110)		p-value
	n	Percentage or Mean (SD)	n	Percentage or Mean (SD)	n	Percentage or Mean (SD)	
Education							0.28
Some high school/high school	1	6.67%	18	24.66%	17	15.45%	
Some college/college	5	33.33%	12	16.44%	20	18.18%	
Graduate	1	6.67%	1	1.37%	6	5.45%	
Unknown	8	53.33%	42	57.53%	67	60.91%	
Race							0.5
White	13	86.67%	63	86.30%	87	79.09%	
Black	1	6.67%	5	6.85%	16	14.54%	
Asian	0	0%	2	2.74%	2	1.82%	
Other/multiracial	1	6.67%	3	4.12%	2	1.82%	
Unknown	0	0%	0	0%	3	2.73%	
Ethnicity							0.86
Hispanic	4	26.67%	17	23.29%	20	18.18%	
Non-Hispanic	11	73.33%	55	75.34%	88	80%	
Unknown	0	0%	1	1.37%	2	1.82%	
Insurance							0.43
Public	4	26.67%	27	36.99%	47	42.73%	
Private	11	73.33%	46	63.01%	63	57.27%	
Pre-pregnancy BMI							0.53
Underweight	0	0%	0	0%	3	2.73%	
Normal	4	26.67%	30	41.1%	34	30.91%	
Overweight	5	33.33%	15	20.55%	28	25.45%	
Obese	6	40%	28	38.36%	45	40.91%	
Rurality							0.91
Metropolitan	1	6.67%	6	8.22%	9	8.18%	
Micropolitan	14	93.33%	64	87.67%	97	88.18%	
Small town	0	0%	2	2.74%	2	1.82%	
Rural	0	0%	1	1.37%	2	1.82%	
Vaccination	6	40%	19	26.03%	20	18.18%	0.12
Tobacco use	1	6.67%	15	20.55%	12	10.91%	0.15
Alcohol use	0	0%	1	1.37%	3	2.73%	0.69
Drug use	0	0%	11	15.07%	18	16.36%	0.24
Age (years)		30.66 (7.04)		29.06 (5.27)		28.69 (6.26)	0.48

Maternal outcomes

Maternal outcomes varied by trimester of infection. Pneumonia was significantly more common in third-trimester cases (13/110, 11.8%, p=0.024; Table 2). There were no significant differences in the rates of gestational diabetes or gestational hypertension/preeclampsia across trimesters. ICU admissions were rare but occurred only among women infected in the third trimester (3/110, 2.7%). No maternal deaths were recorded during the study period.

**Table 2:** Maternal outcomes based on trimester of COVID-19 diagnosis Data are presented as n (%) for categorical variables and mean ± SD for continuous variables. Odds ratios (ORs) or beta coefficients (β) with 95% confidence intervals (CIs) and standard errors (SEs) represent comparisons to third-trimester infections. P-values reflect differences across trimesters. P-values were calculated using chi-square or Fisher’s exact tests for categorical variables and analysis of variance (ANOVA) for continuous variables. Odds ratios (ORs) and beta coefficients (β) were derived from logistic and linear regression models, respectively. Analyses were adjusted for covariates as indicated: ¹Insurance (mode of delivery), ²BMI (gestational diabetes and hypertension), ³Education (weight gain).

Variables	First Trimester (n=15)		Second Trimester (n=73)		Third Trimester (n=110)		p-value	OR (95% CI) or ꞵ (SE; p-value)
	n	Percentage or Mean (SD)	n	Percentage or Mean (SD)	n	Percentage or Mean (SD)		
Mode of delivery^1^							0.45	0.45 (0.24, 0.88)
Cesarean	5	33.33%	26	35.62%	32	29.09%		
Vaginal	10	66.67%	47	64.38%	78	70.91%		
Gestational diabetes^2^	3	20%	13	17.81%	16	14.55%	0.52	0.72 (0.33, 1.58)
Gestational hypertension^2^	4	26.67%	14	19.18%	18	16.36%	0.49	0.77 (0.36, 1.62)
ICU admission	0	0%	0	0%	3	2.73%	0.96	>999.999 (<0.001, >999.999)
Polyhydramnios	0	0%	4	5.48%	5	4.55%	1	1.00 (0/26, 3.84)
Pneumonia	0	0%	2	2.74%	13	11.82%	0.024	5.76 (1.26, 26.26)
Weight gain (kg)^3^		11.79 (7.09)		11.76 (7.86)		10.94 (7.06)	0.4	-0.68 (0.98; 0.48)

Neonatal outcomes

Table 3 presents the characteristics of the 201 newborns delivered to women with COVID-19 by trimester of maternal infection: 17 in the first trimester, 74 in the second, and 110 in the third. Across all trimesters, most newborns were White (82.4% in the first, 86.5% in the second, and 79.1% in the third), while Black newborns represented 5.9%, 6.8%, and 14.6%, respectively. Hispanic ethnicity was reported in 29.4% of first-trimester, 23.0% of second-trimester, and 18.2% of third-trimester newborns.

**Table 3:** Characteristics of newborns delivered to women with COVID-19 by trimester of maternal infection Data are presented as n (%) for categorical variables. P-values indicate differences across trimesters. Race, ethnicity, and rurality of newborns are reported, with the majority being White, non-Hispanic, and from micropolitan areas. P-values were calculated using chi-square or Fisher’s exact tests for categorical variables and analysis of variance (ANOVA) for continuous variables.

Variables	First Trimester (n=17)		Second Trimester (n=74)		Third Trimester (n=110)		p-value
	n	Percentage or Mean (SD)	n	Percentage or Mean (SD)	n	Percentage or Mean (SD)	
Race							0.23
White	14	82.35%	64	86.49%	87	79.09%	
Black	1	5.88%	5	6.76%	16	14.55%	
Asian	0	0%	2	2.70%	2	1.82%	
Other/multiracial	2	11.76%	3	4.05%	2	1.82%	
Unknown	0	0%	0	0%	3	2.73%	
Ethnicity							0.79
Hispanic	5	29.41%	17	22.97%	20	18.18%	
Non-Hispanic	12	70.59%	56	75.68%	88	80%	
Unknown	0	0%	1	2.35%	2	1.82%	
Rurality							0.98
Metropolitan	1	5.88%	6	8.11%	9	8.18%	
Micropolitan	16	94.12%	65	87.84%	97	88.18%	
Small town	0	0%	2	2.70%	2	1.82%	
Rural	0	0%	1	1.35%	2	1.82%	

Neonatal findings demonstrated an association between early infection and adverse outcomes. First-trimester infection was associated with increased risk of preterm birth (7/17, 41.2%; OR 3.83, 95% CI 1.28-11.45) and low birth weight (4/17, 23.5%; OR 11.24, 95% CI 3.04-41.55), as shown in Table 4. Mean gestational age at delivery was lowest among neonates exposed in the first trimester (35 weeks, 4 days) compared with those exposed in the second (38 weeks, 2 days) and third (37 weeks, 6 days) trimesters (p = 0.001). NICU admission rates ranged from 9% to 24% but did not differ significantly by trimester. APGAR scores at one and five minutes were similar across groups.

**Table 4:** Newborn outcomes by trimester of maternal COVID-19 infection Data are presented as n (%) for categorical variables and mean ± SD for continuous variables. Odds ratios (ORs) or beta coefficients (β) with 95% confidence intervals (CIs) and standard errors (SEs) represent comparisons to third-trimester infections. P-values reflect differences across trimesters. P-values were calculated using chi-square or Fisher’s exact tests for categorical variables and analysis of variance (ANOVA) for continuous variables. Odds ratios (ORs) and beta coefficients (β) were derived from logistic and linear regression models, respectively. Analyses were adjusted for covariates as indicated: ¹maternal vaccination (low birth weight, 1-minute APGAR, birthweight) and ²maternal age (stillbirth). NICU: neonatal intensive care unit; APGAR: Appearance, Pulse, Grimace, Activity, Respiration.

Variable	First Trimester (n = 17)	Second Trimester (n = 74)	Third Trimester (n = 110)	p-value	1st vs 3rd OR (95% CI) or β (SE; p)	2nd vs 3rd OR (95% CI) or β (SE; p)
NICU stay	4 (23.53%)	10 (13.51%)	10 (9.09%)	0.22	3.08 (0.842, 11.24)	1.56 (0.62, 3.96)
Preterm	7 (41.18%)	11 (14.86%)	17 (15.45%)	0.044	3.83 (1.28, 11.45)	0.96 (0.42, 2.17)
Low birth weight^1 ^(<2500 g)	7 (23.53%)	6 (8.11%)	10 (9.09%)	0.006	11.24 (3.04, 41.55)	0.96 (0.03, 2.78)
Stillbirth^2^	1 (5.88%)	0 (0%)	3 (2.73%)	0.93	1.56 (0.14, 17.12)	<0.001 (<0.001, >999.99)
1 min APGAR^1^	6.88 ± 2.71	7.94 ± 1.33	7.77 ± 1.98	0.10	−1.07 (0.48; 0.03)	0.11 (0.28; 0.68)
5 min APGAR	8.12 ± 2.23	8.82 ± 0.53	8.55 ± 1.69	0.15	−0.44 (0.38; 0.25)	0.27 (0.22; 0.21)
Birthweight (g)^1^	2849.06 ± 1260.57	3242.58 ± 533.09	3143.72 ± 655.45	0.10	−351.22 (179.01; 0.05)	80.43 (102.50; 0.43)
Gestational age (weeks, days)	35w 4d	38w 3d	37w 6d	0.001	−15.67 (p = 0.002)	3.50 (p = 0.22)

Racial disparities

When outcomes were stratified by race, maternal and neonatal clinical outcomes were broadly similar between Black and White women (Tables 5, 6). However, disparities in structural and social factors were evident. Black women were more likely to rely on public insurance (68% vs. 33%, p = 0.003), less likely to be vaccinated against COVID-19 (5% vs. 25%, p = 0.03), and more frequently reported drug use (36% vs. 12%, p = 0.01).

**Table 5:** Characteristics of delivering women by race (n=185) Data are presented as n (%) for categorical variables and mean ± SD for continuous variables. Comparisons between White and Black women are shown with p-values. P-values were calculated using chi-square or Fisher’s exact tests for categorical variables and analysis of variance (ANOVA) for continuous variables. Statistically significant differences were observed for insurance status, COVID-19 vaccination, and drug use; other variables showed no significant differences.

Variables	White (n=163)		Black (n=22)		p-value
	n	Percentage or Mean (SD)	n	Percentage or Mean (SD)	
Education					0.08
Some high school/high school	28	17.78%	7	31.82%	
Some college/college	34	20.86%	0	0%	
Graduate	7	4.29%	1	4.54%	
Unknown	94	57.67%	14	63.64%	
Ethnicity					0.05
Hispanic	33	20.24%	0	0	
Non-Hispanic	127	77.91%	22	100	
Unknown	3	1.84%	0	0	
Insurance					0.003
Public	54	33.13%	15	68.18%	
Private	109	66.87%	7	31.82%	
Pre-pregnancy BMI					0.71
Underweight	3	1.84%	0	0%	
Normal	56	34.35%	7	31.82%	
Overweight	36	22.08%	7	31.82%	
Obese	68	41.72%	8	36.36%	
Rurality					0.32
Metropolitan	16	9.82%	0	0%	
Micropolitan	140	85.89%	22	100%	
Small town	4	2.45%	0	0%	
Rural	3	1.84%	0	0%	
COVID-19 vaccination					0.03
Vaccinated	41	25.15%	1	4.54%	
Not vaccinated	122	74.85%	21	95.45%	
Tobacco	24	14.72%	3	13.64%	0.89
Alcohol use	4	2.45%	0	0%	0.96
Drug use	20	12.27%	8	36.36%	0.01
Age (years)		29.09 (5.92)		26.95 (5.64)	0.11

**Table 6:** Maternal outcomes by race Data are presented as n (%) for categorical variables and mean ± SD for continuous variables. Odds ratios (OR) or beta coefficients (β) with 95% confidence intervals (CI) and standard errors (SE) compare outcomes between Black and White women. P-values were calculated using chi-square or Fisher’s exact tests for categorical variables and analysis of variance (ANOVA) for continuous variables. Odds ratios (ORs) and beta coefficients (β) were derived from logistic and linear regression models, respectively. P-values indicate statistical significance. No significant differences were observed across measured maternal outcomes.

Variables	White (n=163)		Black (n=22)		p-value	OR (95% CI) or ꞵ (SE)
	n	Percentage or Mean (SD)	n	Percentage or Mean (SD)		
Mode of delivery					0.59	0.76 (0.28, 2.07)
Cesarean	53	32.51%	7	31.82%		
Vaginal	110	67.48%	15	68.18%		
Gestational diabetes	28	17.18%	1	4.55%	0.16	0.22 (0.03, 1.77)
Hypertension gestational/preeclampsia	32	19.63%	2	9.09%	0.23	0.41 (0.09, 1.85)
ICU admission	3	1.84%	0	0%	0.97	<0.001 (<0.001, >999.999)
Polyhydramnios	7	4.29%	1	4.55%	0.96	1.06 (0.12, 9.06)
Pneumonia	11	6.75%	3	13.64%	0.26	2.18 (0.56, 8.53)
Weight gain (kg)		11.34 (7.42)		11.18 (7.00)	0.92	-0.17 (1.75)

County comparisons

Comparison with Winnebago County data is presented in Table 7. Women in the Mercyhealth cohort experienced higher rates of gestational diabetes (16.2% vs. 10.8%, p = 0.02) and ICU admission (1.5% vs. 0.3%, p = 0.01). Rates of cesarean delivery, preterm birth, low birth weight, and NICU admission were similar between the two populations. The Winnebago County 2021 maternal outcomes data, obtained directly from the Illinois Department of Public Health (IDPH), Division of Health Data & Policy, Office of Policy, Planning & Statistics, and provided to our study team by the state Vital Statistician (internal, unpublished data), were used for these comparisons. During the study period, 198 pregnant women were diagnosed with COVID-19 at Mercyhealth. There were 3,283 total deliveries in Winnebago County in 2021. Odds ratios were used to analyze the data, with statistical significance defined as a p-value ≤ 0.05.

**Table 7:** Maternal and neonatal outcomes at Mercyhealth vs. Winnebago County Data are presented as percentages for categorical variables. Odds ratios (OR) with 95% confidence intervals (CI) compare outcomes between Mercyhealth and county-wide deliveries. P-values indicate statistical significance. Mercyhealth patients had higher rates of gestational diabetes and ICU admission, while hypertension was more common county-wide; other outcomes showed no significant differences. P-values were calculated using chi-square or Fisher’s exact tests for categorical variables and analysis of variance (ANOVA) for continuous variables. Odds ratios (ORs) were derived from logistic regression models.

Variables	Mercyhealth	Winnebago	p-value	OR (95% CI)
Gestational diabetes	16.16%	10.84%	0.02	1.58 (1.07, 2.35)
Hypertension diagnosis (gestational hypertension and/or pre-eclampsia)	18.18%	44.78%	<0.0001	4.74 (3.19, 7.05)
Cesarean delivery	31.82%	36.61%	0.17	0.81 (0.59, 1.10)
ICU admission	1.51%	0.27%	0.01	5.60 (1.50, 20.84)
Preterm birth	17.40%	13.37%	0.11	1.36 (0.94, 1.99)
NICU admission	11.94%	12.58%	0.59	0.89 (0.57, 1.37)
Low birth weight	11.44%	10.39%	0.63	1.11 (0.71, 1.74)

## Discussion

This study examined maternal and neonatal outcomes, racial disparities, and county-level comparisons among pregnant women with COVID-19 at a single institution in Rockford, Illinois. Our findings suggest that the trimester of infection may influence outcomes, with maternal pneumonia more frequent in the third trimester and adverse neonatal outcomes more common following first-trimester infection. These trimester-specific associations are biologically plausible given the physiological and immunological changes that occur throughout pregnancy.

The increased risk of pneumonia observed in third-trimester infections is consistent with prior reports demonstrating heightened respiratory vulnerability late in gestation. Physiological changes, including reduced pulmonary reserve, elevated oxygen demand, and immunologic adaptations, likely contribute to this increased risk [9,10]. Similarly, the association between first-trimester infection and preterm birth or low birth weight is consistent with emerging evidence that early maternal infection may impair placental development and vascular function, resulting in downstream effects on fetal growth and gestational length [11]. While these associations are supported by prior literature, the small first-trimester subgroup underscores the need for cautious interpretation and highlights the importance of validating these findings in larger cohorts.

When examining outcomes by race, our results showed no significant differences in maternal or neonatal clinical outcomes between Black and White women. However, disparities were evident in social and structural factors, including insurance coverage, vaccination uptake, and reported substance use. These findings reflect broader systemic inequities in maternal healthcare access and delivery, including disparities in prenatal care access, insurance coverage, and healthcare utilization. Prior studies have demonstrated reduced adequacy and delayed initiation of prenatal care among Black pregnant patients during the COVID-19 pandemic, as well as disparities in COVID-19 infection risk in pregnancy [7,11]. Although clinical outcomes were comparable in this limited cohort, the underlying inequities remain relevant for future research and policy interventions.

Comparison with Winnebago County maternal outcomes data provided additional context. Our institution-level results were evaluated alongside trends reported in the Winnebago County 2021 maternal outcomes data, obtained directly from the Illinois Department of Public Health (IDPH), Division of Health Data & Policy, Office of Policy, Planning & Statistics. These data, provided by the state Vital Statistician, offer important contextual benchmarks but are not publicly published or available in a citable format. Overall, the consistency between our findings and the county-level statistics supports the interpretation of our results while highlighting potential areas for targeted, equity-focused interventions. Women with COVID-19 at Mercyhealth demonstrated higher rates of gestational diabetes and ICU admission compared with the county population, whereas rates of cesarean delivery, preterm birth, and NICU admission were similar. Non-pharmacologic interventions, particularly structured dietary modification and physical activity, are first-line components of gestational diabetes management and have been shown to improve glycemic control and reduce adverse pregnancy outcomes. Recent evidence suggests that even among patients with elevated fasting glucose levels, targeted lifestyle interventions can improve maternal metabolic parameters and neonatal outcomes [12]. Although these factors were not captured in our dataset, they represent important modifiable contributors that may partially explain differences in gestational diabetes rates and should be considered in future studies.

Mercyhealth serves as a tertiary care center with a Level III NICU and is the regional perinatal center, meaning some patients represented higher-acuity referrals or transports from surrounding hospitals. Consequently, observed differences in certain outcomes may reflect referral patterns and case mix, in addition to the impact of COVID-19. While COVID-19 may contribute to increased metabolic and critical care burdens, not all maternal or neonatal outcomes differ substantially from the general population, highlighting the complex interplay between infection, preexisting risk factors, and health system characteristics.

Several limitations should be noted. This single-center study with a modest sample size may limit generalizability. Subgroup analyses, particularly for first-trimester infections and race-stratified comparisons, were underpowered and should be interpreted as preliminary. Sparse events (ICU admission, pneumonia) contributed to wide confidence intervals and potential model instability. Trimester classification was based on the timing of diagnosis rather than confirmed infection onset, which may introduce exposure misclassification. COVID-19 severity was not formally classified, limiting the assessment of outcomes by disease severity. Analyses were not adjusted for multiple comparisons, increasing the risk of type I error. The absence of a contemporaneous COVID-negative control group limits causal inference, and differences in population characteristics and data definitions may limit comparability with county-level data. Overall, findings should be interpreted as exploratory and hypothesis-generating.

Despite these limitations, this study contributes important local evidence on COVID-19 in pregnancy. By examining outcomes across trimesters, evaluating racial disparities, and contextualizing findings with county-level data, the study provides a comprehensive perspective that can inform prenatal counseling and clinical care. The results emphasize the importance of trimester-specific monitoring, particularly for respiratory complications in late pregnancy and fetal growth in early pregnancy, and highlight the ongoing need to address disparities in prenatal care access, insurance coverage, and COVID-19 vaccination uptake in maternal healthcare. Future work should build on these findings through larger, multi-center studies and long-term follow-up of infants exposed to maternal COVID-19 in utero.

## Conclusions

This single-center cohort suggests trimester-specific differences in maternal and neonatal outcomes among pregnancies complicated by COVID-19. Third-trimester infection was associated with a higher incidence of maternal pneumonia, while first-trimester infection was associated with increased rates of preterm birth and low birth weight. Although clinical outcomes were broadly similar by race, disparities in vaccination status, insurance coverage, and reported substance use highlight persistent inequities in maternal healthcare. Comparison with county-level data demonstrated higher rates of gestational diabetes and ICU admission in the study cohort, which may reflect both the impact of COVID-19 and the tertiary referral nature of our institution. These findings provide locally relevant, exploratory evidence to inform counseling and clinical monitoring, and support equity-focused interventions, including promoting COVID-19 vaccination in pregnancy as a key strategy to reduce adverse maternal and neonatal outcomes. However, larger, multi-center studies are needed to confirm these associations and assess long-term outcomes.
